# Cost-effectiveness analysis for rotavirus vaccine decision-making: How can we best inform evolving and complex choices in vaccine product selection?

**DOI:** 10.1016/j.vaccine.2019.12.014

**Published:** 2020-02-05

**Authors:** Clint Pecenka, Frederic Debellut, Naor Bar-Zeev, Palwasha Anwari, Justice Nonvignon, Andrew Clark

**Affiliations:** a PATH, 2201 Westlake Ave, Suite 200, Seattle, WA 98121, USA; b PATH, Rue de Varembé 7, 1202 Geneva, Switzerland; c International Vaccine Access Center, Department of International Health, Johns Hopkins Bloomberg School of Public Health, 615 N. Wolfe Street, Baltimore, MD 21205, USA; d Malawi-Liverpool-Wellcome Trust Clinical Research Programme, College of Medicine, University of Malawi, Blantyre, Malawi; e Afghanistan National Immunization Technical Advisory Group, District 10, Kabul, Afghanistan; f School of Public Health, University of Ghana, Legon, Ghana; g London School of Hygiene and Tropical Medicine, Keppel Street, London WC1E 7HT, United Kingdom

Rotavirus vaccination has been a global health success story. In 2008, RotaTeq® received World Health Organization (WHO) prequalification, which was followed by the prequalification of ROTARIX® in 2009 [1]. Over the following decade, these two vaccines were introduced in around 100 countries’ immunization programs and have contributed to significant declines of the global rotavirus disease burden [2], [3], [4], [5]. Then, in 2018, two new rotavirus vaccines made in India, ROTAVAC® and ROTASIIL®, also received WHO prequalification. This was a landmark achievement for the rotavirus community, as it expands product choice and has the potential to help lower prices, mitigate supply concerns, and enhance rotavirus vaccine impact around the world through the broader use of rotavirus vaccines, particularly in low- and middle-income countries. However, having a range of rotavirus vaccine options with heterogeneous characteristics including different presentations, dosing schedules, and prices can also present decision-makers with more complex choices in selecting a vaccine product. This challenge is likely most pressing in countries where rotavirus burden is highest, resources are particularly constrained, and the feasibility of fully assessing and comparing all options is most limited.

Gavi, the Vaccine Alliance, and international partners recognized this challenge and have been working to compile information to help country decision-makers evaluate the available rotavirus vaccine products [1]. These resources include details on the vaccines’ efficacy, effectiveness, safety, cost, cost-effectiveness, storage and transportation requirements, and other programmatic considerations. Despite considerable effort, decision-making remains challenging for those seeking to optimize rotavirus vaccine product choices. Reasons for this difficulty may include the breadth and depth of the available information, challenges in prioritizing different product attributes, the dynamic nature of the rotavirus vaccine market, the lack of guidance on when and how to consider product switches and in some cases, interpretation of the available information.

Cost and cost-effectiveness are important considerations in making rotavirus vaccine product choices; they also exemplify some of the challenges that countries face. A recent publication in this journal (written by the authors of this commentary) compared the costs and cost-effectiveness of three alternative rotavirus vaccines in three countries supported by Gavi (Bangladesh, Ghana, and Malawi), assuming common efficacy in each country across the three products [6]. The analysis concluded that all three rotavirus vaccines examined were likely to be cost-effective in each country compared to no vaccination. In addition, one of the products was consistently the least costly and most cost-effective of the three examined (Table 1). While this finding was consistent across countries, it was also reported as “sensitive to relatively modest changes” in vaccine prices or vaccine delivery costs. In short, the previously published analysis showed that: (1) all three vaccines were likely cost-effective compared to no vaccination, (2) ROTARIX was the least costly and most cost-effective product in the three examined Gavi-supported countries, and (3) the results were consistent but sensitive to small changes in input values, in particular where there is significant uncertainty (e.g. vaccine prices or vaccine delivery costs).

**Table 1:** Cost and cost-effectiveness by country and vaccine product, initial analysis.

	Bangladesh	Ghana	Malawi
Cost per DALY* averted (country perspective, with Gavi subsidy)	$61 (ROTARIX); $153 (ROTAVAC); $216 (ROTASIIL)	$230 (ROTARIX); $283 (ROTAVAC); $358 (ROTASIIL)	$7 (ROTARIX); $38 (ROTAVAC); $32 (ROTASIIL)
Total country cost of vaccination program with Gavi subsidy**	$41.6 M (ROTARIX); $53.5 M (ROTAVAC); $61.7 M (ROTASIIL)	$67.9 M (ROTARIX); $81.5 M (ROTAVAC); $100.5 M (ROTASIIL)	$10.2 M (ROTARIX); $14.5 M (ROTAVAC); $13.5 M (ROTASIIL)
Least costly and most cost-effective product	ROTARIX	ROTARIX	ROTARIX

* Disability adjusted life year.

** All cost and cost-effectiveness estimates in this article incorporate wastage rates as previously reported [6].

In less than a year since the publication of this cost-effectiveness analysis, the reported prices for all three vaccines have changed, some dramatically. In addition, new information is emerging on the delivery costs (e.g. administration, cold chain, training) for the new rotavirus vaccines. While the initial analysis remains an accurate portrayal of the world at that time and illustrated key uncertainties that would influence findings, the results no longer accurately reflect the choices now facing these three countries. In fact, if the initial analysis had been undertaken with today’s best information, all vaccines would remain cost-effective compared to no vaccination, but the rank order might now be very different.

Table 2 illustrates two additional scenarios using the previously reported modelling approach and data inputs [6]. We again assume common efficacy values across products in each country as we do not believe there is yet enough evidence to differentiate by product. Scenario 1 incorporates updated pricing data for ROTAVAC (five-dose vial, frozen presentation) and ROTASIIL (two-dose vial, lyophilized presentation) and emerging information from a single setting outside of our examined countries which suggests that delivery costs for ROTAVAC are lower than those for ROTARIX by approximately $0.30, primarily due to lower cold chain costs [1], [7]. Scenario 2 incorporates these changes and assumes that emerging findings on ROTAVAC delivery costs could also apply to ROTASIIL. While there are some data to support these scenarios, evidence remains limited. As such, Table 2 is meant to illustrate the sensitivity of the results to relatively small changes to input parameters rather than a clear argument for a single, economically preferred product across these countries.

**Table 2:** Cost and cost-effectiveness by country and vaccine product, subsequent analysis.

	Bangladesh	Ghana	Malawi
**Scenario 1: Updated vaccine prices; applying lower delivery costs for ROTAVAC**			
Cost per DALY averted (country perspective, with Gavi subsidy)	$76 (ROTARIX); Cost-saving (ROTAVAC); $126 (ROTASIIL)	$249 (ROTARIX); $220 (ROTAVAC); $251 (ROTASIIL)	$7 (ROTARIX); Cost-saving (ROTAVAC); $32 (ROTASIIL)
Total country cost of vaccination program with Gavi subsidy	$43.6 M (ROTARIX); $29.5 M (ROTAVAC); $50.0 M (ROTASIIL)	$72.6 M (ROTARIX); $65.3 M (ROTAVAC); $73.3 M (ROTASIIL)	$10.2 M (ROTARIX); $7.3 M (ROTAVAC); $13.5 M (ROTASIIL)
Least costly and most cost-effective product	ROTAVAC	ROTAVAC	ROTAVAC

**Scenario 2: Updated vaccine prices; applying lower delivery costs for ROTAVAC and ROTASIIL**			
Cost per DALY averted (country perspective, with Gavi subsidy)	$76 (ROTARIX); Cost-saving (ROTAVAC); Cost-saving (ROTASIIL)	$249 (ROTARIX); $220 (ROTAVAC); $207 (ROTASIIL)	$7 (ROTARIX); Cost-saving (ROTAVAC); Cost-saving (ROTASIIL)
Total country cost of vaccination program with Gavi subsidy	$43.6 M (ROTARIX); $29.5 M (ROTAVAC); $28.0 M (ROTASIIL)	$72.6 M (ROTARIX); $65.3 M (ROTAVAC); $62.0 M (ROTASIIL)	$10.2 M (ROTARIX); $7.3 M (ROTAVAC); $6.4 M (ROTASIIL)
Least costly and most cost-effective product	ROTASIIL	ROTASIIL	ROTASIIL

There are two important findings from Scenario 1. First, ROTAVAC is now projected to be cost-saving in two of the three countries examined. This means the cost of averted illnesses exceeds the cost of the vaccination program, so the vaccination program both enhances health and saves money. Second, ROTAVAC is the least costly and most cost-effective product in all countries in this scenario, a result distinct from the previously published analysis. Scenario 2, however, shows ROTASIIL to also be cost-saving in two of three countries and suggests ROTASIIL is the least costly and most cost-effective product in all three countries. In short, the economically preferred vaccine product is sensitive and subject to change across relatively similar scenarios.

One might conclude from this revised analysis that economic information does not enhance rotavirus vaccine product decisions because the economically preferred product is so sensitive small changes in key inputs. That interpretation, however, overlooks the utility of using economic evaluation to identify these sensitive parameters and millions of dollars that a country might save through the selection of an economically preferred product. We argue that economic considerations remain critical to product choice but require better data on delivery costs and careful consideration of key tradeoffs, especially the interaction of vaccine price, incremental delivery costs, and the number of doses per course. It is likely that the least costly product in one country may be the most expensive for a neighboring country, but we might also see one product favored by groups of countries. For example, countries ineligible for Gavi support might favor one product over others and we may see different trends in the Gavi market.

This commentary avoids specific guidance to countries seeking to select an economically preferred product but offers a few thoughts. First, economic considerations should continue to be part of a product selection criteria and can help identify critical uncertainties that influence results. As this discussion demonstrates, these choices are not simple, but international partners can help provide impartial perspective and experience. Second, any of the prequalified rotavirus vaccines are likely to be cost-effective in most countries. Assuming products under consideration are affordable, which should be assessed, any of the prequalified products are likely to be good economic choices. However, the “best” choice may be highly sensitive to country context including vaccine prices and delivery costs. Third, a periodic assessment of current product availability, product characteristics, and prices can help ensure an effective, affordable, and sustainable rotavirus vaccination program.

## Declaration of Competing Interest

The authors declare that they have no known competing financial interests or personal relationships that could have appeared to influence the work reported in this paper.
